# Alternative polyadenylation signals and promoters act in concert to control tissue-specific expression of the Opitz Syndrome gene *MID1*

**DOI:** 10.1186/1471-2199-8-105

**Published:** 2007-11-15

**Authors:** Jennifer Winter, Melanie Kunath, Stefan Roepcke, Sven Krause, Rainer Schneider, Susann Schweiger

**Affiliations:** 1 Max-Planck Institute for Molecular Genetics, Berlin-Dahlem, Germany; 2 Institute of Biochemistry, University Innsbruck, Austria; 3 ALTANA Pharma AG, Preclinical Research Bioinformatics, Konstanz, Germany; 4 Department of Dermatology, Charité-Hospital, Berlin, Germany; 5 Department of Neuroscience and Pathology, College of Medicine, University of Dundee, Dundee, UK

## Abstract

**Background:**

Mutations in the X-linked *MID1 *gene are responsible for Opitz G/BBB syndrome, a malformation disorder of developing midline structures. Previous Northern blot analyses revealed the existence of at least three *MID1 *transcripts of differing lengths.

**Results:**

Here we show that alternative polyadenylation generates the size differences observed in the Northern blot analyses. Analysis of EST data together with additional Northern blot analyses proved tissue-specific usage of the alternative polyadenylation sites. Bioinformatic characterization of the different 3'UTRs of *MID1 *revealed numerous RNA-protein interaction motifs, several of which turned out to be conserved between different species. Furthermore, our data suggest that mRNA termination at different polyadenylation sites is predetermined by the choice of alternative 5'UTRs and promoters of the *MID1 *gene, a mechanism that efficiently allows synergistic function of 5' and 3'UTRs.

**Conclusion:**

*MID1 *expression is tightly regulated through concerted action of alternative promoters and alternative polyadenylation signals both during embryonic development and in the adult.

## Background

Mutations in the X-linked *MID1 *gene cause Opitz G/BBB syndrome (OS). OS is a congenital malformation syndrome characterized by defective ventral midline development with the main features being ocular hypertelorism and hypospadias. Additional abnormalities such as cleft lip and palate, laryngo-tracheal fistulas, heart defects, imperforate anus and mental retardation may also be present.

Recently we found that the MID1 protein associates with microtubules [1] and triggers ubiquitination and degradation of the microtubule-associated protein phosphatase 2a (PP2A) upon interaction with the α4 protein [2]. MID1 loss-of-function mutations, as seen in OS patients, thus cause accumulation of microtubule-associated PP2A and hypophosphorylation of its target proteins.

The *MID1 *mRNA is subject to extensive alternative splicing [3]. Also, several 5'-untranslated regions have been identified and the use of five alternative promoters results in the production of additional *MID1 *transcript isoforms [4].

The expression pattern of *MID1 *has been investigated by Northern blot analyses and in situ hybridization [5-8]. In humans, three transcripts of ~7 kb, ~4.5 kb and ~3.5 kb were observed in all fetal and adult tissues analyzed [6,9]. Remarkably, the coding sequence of *MID1 *accounts for only ~2 kb, and the size differences between the known *MID1 *sequence and the transcripts cannot be explained by alternative splicing of either the coding region or 5'UTR. However, splicing and/or alternative polyadenylation of the 3'UTR have not been investigated so far.

The 3'UTRs of many genes have been shown to be involved in pleiotropic regulatory functions, such as RNA localization, mRNA degradation and stabilization, and translational control. In the present work we describe the identification of several alternative polyadenylation sites in the human *MID1 *3'UTRs which give rise to transcripts with four different 3'UTRs and tissue-specific expression patterns.

To identify putative regulatory structures we have characterized the *MID1 *3'UTR with bioinformatic tools and report the presence of putative target sites for RNA binding proteins. Notably, we identified several AU-rich elements (AREs) and cytoplasmic polyadenylation elements (CPEs). As proteins binding to both AREs and CPEs are known to be key regulators of mRNA stability and/or translation, our results suggest a tight control of MID1 expression through the different 3'UTRs. Intriguingly, we also found that specific polyadenylation signals are arrayed with distinct 5'UTRs and promoters of the *MID1 *gene, indicating that polyadenylation is a promoter-driven process.

## Results

### EST data indicate alternative polyadenylation of the MID1 gene

**Figure 1:**
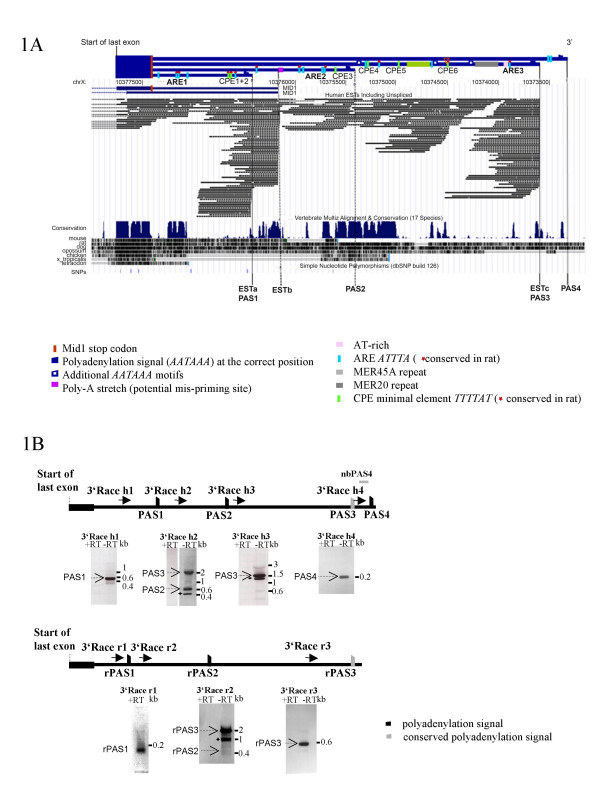
Alternative polyadenylation sites in the *MID1 *mRNAs of human (PAS1–PAS4) and rat (rPAS1–rPAS3). (A) The 3'UTR of human *MID1 *containing alternative polyadenylation sites identified in this study together with data on mRNAs, ESTs and conservation obtained from the UCSC genome browser March 2006 assembly. Regulatory motifs are highlighted in different colors. (B) Ethidium bromide gels of 3'RACE experiments. Locations of primers are indicated in the cartoons (arrows). Asterisks indicate unspecific products. PAS3 and rPAS3 are homologues. The nucleotide sequences of novel 3'ends have been submitted to Genbank with accession numbers EF217423, EF217424, EF217425, EF217426, EF217427, EF217428, and EF217429. The position of the northern probe nbPAS4 is indicated.

Previous Northern blot analyses of human PolyA^+ ^RNA showed *MID1 *transcripts of ~7 kb, ~4.5 kb and ~3.5 kb [6,9]. As these size differences cannot be explained by alternative splicing of the coding sequence or the 5'UTR, we hypothesized the existence of alternative polyadenylation sites (poly(A) sites) in the 3'UTR. To test this hypothesis we analyzed human EST data overlapping the *MID1 *3'UTR. A review of the human EST database indicated at least three alternative poly(A) sites (Fig. 1a), which we named ESTa, b and c. Whereas ESTa and c contain consensus polyadenylation signals at their 3'ends and therefore seem to terminate at real polyadenylation sites, ESTb does not contain such a signal. A stretch of oligo-A present at the 3'end of ESTb pointed to putative mis-priming of polyT-primers as a likely cause of this artifactual polyadenylation site (Fig. 1a). While 53 ESTs overlap ESTc, only 23 ESTs correspond to ESTa (see additional file 1); this likely reflects preferential use of the polyadenylation site corresponding to ESTc.

### Alternative polyadenylation of the MID1 gene in different species

To confirm alternative polyadenylation experimentally we performed 3'RACE with cDNA derived from human fibroblasts (Fig 1b). Fibroblasts were chosen for this analysis because they express *MID1 *at a moderate level. Interestingly, sequencing of the PCR products revealed four different polyadenylation sites which we named PAS1–4 (Fig. 1a and 1b). Two of them match the EST data: PAS1 corresponded to ESTa and PAS3 to ESTc; however, no EST data were available for PAS2 and PAS4 (Fig. 1a).

Polyadenylation signals consisting of an upstream element (AAUAAA) and a downstream U-rich or GU-rich element are in close proximity to all four poly(A) sites (PAS1–PAS4, see additional file 2), and sequence comparison showed that all four upstream elements are conserved between human and dog. In contrast only some of the human upstream elements are conserved in other species. Whereas the element upstream of PAS2 is conserved between human, opossum and chicken, the element upstream of PAS3 is conserved between human and rat (see additional file 3). To test experimentally whether the *MID1 *mRNA is alternatively polyadenylated in other species we performed 3'RACE on cDNA from rat brain, a tissue known to express high levels of *MID1 *(Fig. 1b). Sequencing of the PCR products revealed three alternative polyadenylation sites, rPAS1–3 (Fig. 1b) with rPAS3 corresponding to PAS3 of the human *MID1 *gene, and rPAS1 and rPAS2 probably representing species-specific sites.

### 3'UTR 4 directs expression of tissue specific transcripts

**Figure 2:**
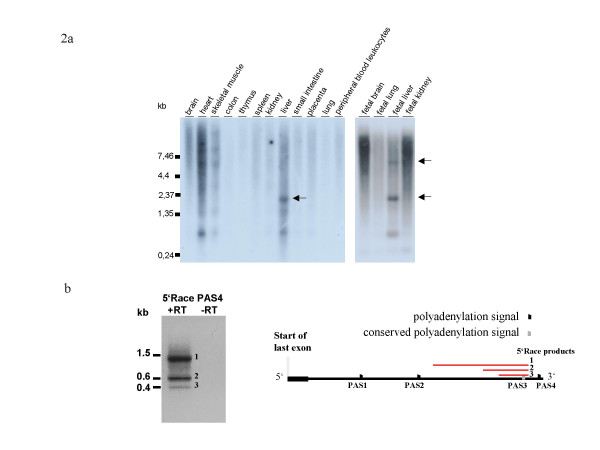
**Northern blot and 5'RACE analysis of human 3'UTR4**. (A) Fetal and adult multiple-tissue Northern blots hybridized with a riboprobe detecting a region between PAS3 and PAS4 of the human 3'UTR. Arrows indicate *MID1 *transcripts. (B) 5'RACE products obtained with primers located downstream of PAS3. The nucleotide sequences of 5'RACE products have been submitted to Genbank with accession numbers EF532594, EF532595, EF532596.

To test for expression of the human transcript terminated by PAS4, we hybridized a specific riboprobe (nbPAS4; Fig. 1b) against commercially available Northern blots containing human polyA^+ ^RNA extracted from a variety of fetal and adult tissues. In contrast to the picture obtained with a probe detecting the *MID1 *open reading frame, which showed ubiquitous expression of a ~7 kb transcript (Quaderi et al. 1997), a transcript of similar size could only be observed in fetal liver and skeletal muscle with nbPAS4 (Fig. 2a, arrows). Additionally, a variety of shorter and longer transcripts were detected in heart, skeletal muscle, liver and fetal liver. Among them a ~2 kb transcript was identified in both adult and fetal liver, making it solely a liver-specific transcript (Fig. 2a, arrow). To further characterize these transcripts we performed 5'RACE experiments on cDNA derived from human fetal liver with a gene-specific primer located downstream of PAS3 (Fig. 2b). Interestingly, sequencing of the PCR products revealed three unspliced transcripts of different lengths with transcription start sites located in the 3'UTR region upstream of PAS3 (Fig. 2b).

### MID1 transcripts starting from alternative 5'UTRs end at specific polyadenylation sites

**Figure 3:**
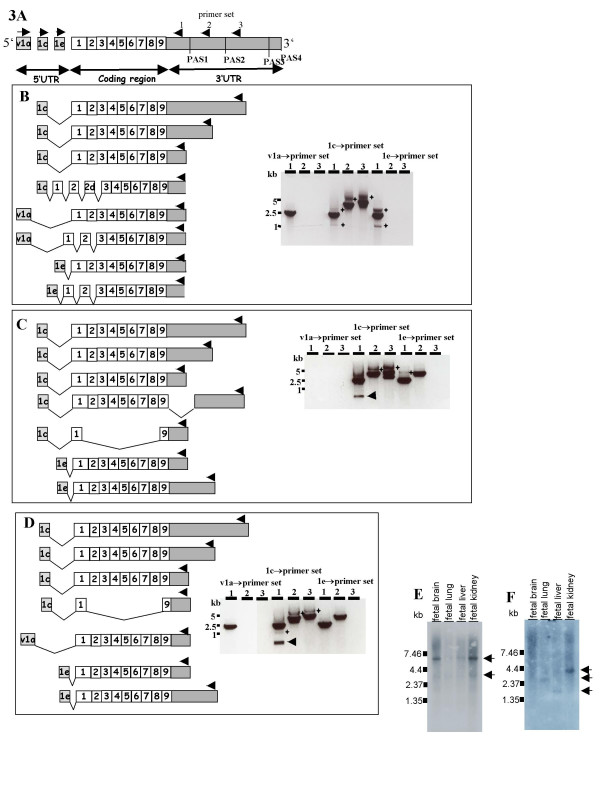
**Identification of full-length *MID1 *transcripts**. (A) 5'UTR exons, coding region and 3'UTR of *MID1*. Arrows indicate location of primers. (B-D) Ethidium bromide stained gels of RT-PCR products obtained using RNA from fibroblasts (B), testis (C) or fetal brain (D). Asterisks indicate products that could not be sequenced due to their low abundance. RT-PCR products obtained with combinations of primer sets 1–3, with primers located in 5'UTR exons 1c, v1a or 1e. (E, F) Fetal tissue Northern blots hybridized with probes corresponding to 5'UTR exons 1c (E) or 1e (F). Arrows indicate *MID1 *transcripts.

Previous Northern blot analyses showed *MID1 *transcripts of ~7 kb, ~4.5 b and ~3.5 kb [6,9], thus indicating that all alternative polyadenylation signals are connected to the full-length coding sequence. However, as human *MID1 *has five alternative promoters and 5'UTRs [4], we tested for preferential and regulated choice of polyadenylation signals in transcripts starting from alternative promoters. RT-PCR experiments were performed using RNA derived from human fibroblasts with primers connecting alternative 5'UTR exons 1a, 1c and 1e to regions upstream of PAS1 (primer set 1), PAS2 (primer set 2) and PAS3 (primer set 3) (Fig. 3a and 3b). Of note, primer set 1 amplified transcripts with poly(A) tails at PAS1–4 whereas primer set 2 amplified transcripts with poly(A) tails at PAS2–4 and primer set 3 exclusively amplified transcripts with poly(A) tails at PAS3–4. For sequencing, PCR products were excised from the gel and cloned into the pGEM-T Easy vector.

Concerning exon 1c, products were amplified with each of the different 3'UTR primer sets (Fig. 3b). In view of the tissue-restricted expression of PAS4, this result clearly shows that transcripts starting in exon 1c are polyadenylated at PAS3 and thus correspond to the 7 kb transcript seen on Northern blots. However, it remains unclear whether there actually are transcripts that start in exon 1c and terminate at PAS1 or PAS2. Sequencing of the cloned PCR products revealed the constitutive *MID1 *coding sequence to be present in five out of six clones (Fig. 3b). One clone represented an in-frame splice variant containing the short variant of exon 1, alternatively spliced exon 2d and constitutive exons 2–9 [3] (Fig. 3b). Because of the comparable sizes of the two transcripts, the RT-PCR products of the alternative splice variant and the constitutive transcript could not be separated on the agarose gel.

RT-PCR with primers located in Exv1a amplified products only in combination with reverse primers located upstream of PAS1, indicating preferential polyadenylation at PAS1 in transcripts derived from promoter 1a (Fig. 3b). However, as the primers located upstream of PAS2 showed inconsistent results in other experiments (see below), we cannot exclude the use of PAS2 with Exv1a. Again, sequencing of three clones confirmed the specificity of the RT-PCR reaction and revealed the presence of the constitutive coding sequence in two of them and the presence of an alternative splice variant containing the short variant of exon 1 in the third (Fig. 3b).

The use of forward primers located in exon 1e led to inconsistent results. In two out of three independent experiments we obtained products when using reverse primers located upstream of PAS3. While we were not able to clone these products, the transcript sizes indicated the presence of the entire 3'UTR sequence in those transcripts. However, when we used reverse primers located upstream of PAS2, no products were obtained in any of the experiments. RT-PCR with reverse primers located upstream of PAS1 amplified transcripts in every experiment (Fig. 3b). Characterization of these transcripts revealed the presence of the constitutive coding sequence in five out of six sequenced clones and a splice variant containing the short variant of exon 1 in the sixth (Fig. 3b).

Our RT-PCR experiments indicate preferential and regulated choice of polyadenylation signals for transcripts starting from each single MID1 promoter. To test whether this phenomenon is a characteristic of fibroblasts or a general regulatory mechanism of *MID1 *expression we performed RT-PCR experiments using RNA derived from two additional human tissues, namely testis and fetal brain (Fig. 3c and 3d). Again, we obtained products with each of the different 3'UTR primer sets when forward primers were located in exon 1c (Fig. 3c and 3d) and only obtained products from fetal brain with primer set 1 when forward primers were located in Exv1a (Fig. 3d). When we used RNA from testis we couldn't obtain any products with primers located in Exv1a indicating that Exv1a is not expressed in this tissue. With exon 1e primers products were amplified only with primer sets 1 and 2 indicating termination at PAS2 in these tissues.

Sequencing of the two cloned products revealed the constitutive *MID1 *coding sequence when primers where located in Exv1a or exon 1e. Concerning exon 1c we additionally obtained two alternative splice variants (Fig. 3c and 3d). One short variant, which was present in both tissues, testis and fetal brain (Fig. 3c and 3d, arrows), contained constitutive exons 1 and 9. The second splice variant lacked part of the 3'UTR but contained the whole constitutive coding region.

In confirmation of the RT-PCR experiments, probes specifically detecting exon 1c or exon 1e were hybridized against commercially available Northern blots containing polyA^+ ^RNA extracted from a variety of human fetal tissues (Fig. 3e and 3f). A ~7 kb and a weaker ~3.5 kb transcript were detected in all fetal tissues analyzed using a probe hybridizing to exon 1c (Fig. 3e) confirming termination of exon 1c transcripts at PAS3 (predominantly) and PAS1. In contrast, a probe hybridizing to exon 1e detected a ~3.5 kb transcript but not a ~7 kb transcript (Fig. 3f). This hybridization pattern indicated that the 7 kb transcript, which would use the PAS3 polyadenylation signal, is a rare mRNA when transcription is initiated by use of promoter e. However, while expression of the ~3.5 kb transcript was high in fetal kidney, weak expression of the same transcript could be detected in the other fetal tissues by autoradiography prior to the final washing of the membrane (data not shown). Also we observed expression of smaller transcripts of ~2.5 kb in fetal lung and ~1.5 kb in fetal liver, suggesting that promoter e drives expression of smaller splice variants in addition to the expression of the constitutive *MID1 *coding sequence (Fig. 3f). However, because hybridization was carried out with a double-stranded DNA-probe, these smaller transcripts might also be overlapping antisense transcripts. Remarkably, in contrast to our RT-PCR experiments we could not detect any transcripts in fetal brain, which indicates their low expression. By direct comparison of the two Northern blots (Fig. 3e and 3f) the two main transcripts of each promoter variant (7 kb when exon 1c is used and 3.5 kb when exon 1 e is used) both appear to be highly expressed in fetal kidney while expression levels of these transcripts and those of smaller sizes appear to vary in all other tissues.

### The MID1 3'UTR contains highly conserved regulatory motifs, which are bound by interacting proteins

In order to screen for functionally relevant sequences within the 3'UTR of *MID1*, evolutionary conservation between human, rat and dog *MID1 *3'UTRs was analyzed. While the overall sequence identity (from the end of the coding sequence through to PAS3 in the rat and through to PAS4 in the dog) is 76% between human and rat and 80% between human and dog, some blocks of stronger sequence similarity are present – the strongest starting 1,852 bp 3' of the translational stop codon, spanning 503 bp, and having a sequence similarity of 88% between human and rat. Parts of the 3'UTR are conserved even between human/rat and more distantly related species like *Xenopus tropicalis *and *Tetraodon *(Fig. 1a), indicating that the *MID1 *3'UTR is under strong selective pressure.

**Figure 4:**
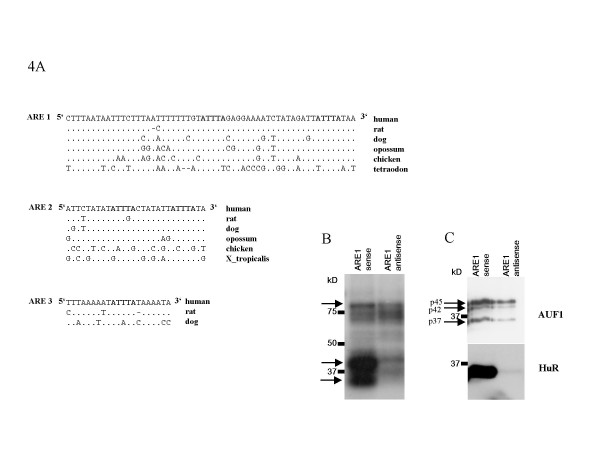
**Regulatory motifs in the 3'UTR of the human *MID1 *gene**. (A) Sequences of ARE motifs 1–3 with surrounding AU-rich sequences. (B) UV-crosslink with protein lysate from HeLa cells and the ARE1 motif. Arrows indicate proteins that bound the ARE1 sense transcript with much higher affinity than the antisense negative control. (C) Western blot analyses of RNA-protein pulldowns with protein lysate from HeLa cells and the ARE1 motif. HuR and AUF1 are detected using specific antibodies.

Conservation of the sequence of the *MID1 *3'UTR suggests the presence of regulatory motifs, such as for the binding of proteins. Bioinformatic analysis indeed identified several putative protein-binding motifs. In addition to motifs like cytoplasmic polyadenylation elements with the consensus sequence TTTTAT [10] and additional polyadenylation signals, we found AU-rich elements (AREs) with the sequence ATTTA in all parts of the 3'UTR (Fig. 1a, 4a and additional file 4). Some of these short ARE motifs were found to be parts of much longer AU-rich sequences which may indicate their functional relevance [11] (Fig. 4a). AREs have been shown to influence RNA stability and/or to control translation of a number of genes [11-13].

Particularly ARE1 seemed to be of potential functional relevance because it comprises a long AU-rich sequence, which is highly conserved in various species (Fig. 4a). To test for binding of interacting proteins to this motif a radioactively labelled transcript corresponding to ARE1 was incubated with HeLa cell lysate and subsequent UV-crosslinking was performed. Complexes were resolved by electrophoresis through SDS acrylamide gels and dried gels were exposed to X-ray film. Interestingly, this method identified several proteins of ~78 and 30–45 kD that had bound the sense ARE1 transcript but not the antisense control (Fig. 4b). In a next step we tried to identify proteins which interact with the ARE1 motif. As candidates we considered the ARE-binding proteins HuR and AUF1 because they have sizes between 32 and 45 kD which correspond to those of the proteins seen in the UV-assay. To test for binding of the candidate proteins we performed a RNA-protein pulldown assay by incubating a biotin labelled transcript corresponding to ARE1 with HeLa cell lysate. RNA-protein complexes were subsequently pulled down with streptavidin magnetic beads. Complexes were resolved by electrophoresis through SDS acrylamide gels. Western blot analyses with specific antibodies directed against AUF1 and HuR showed a clear binding of both proteins to the ARE1 sense RNA, while only little AUF1 protein and almost no HuR had bound to the ARE1 antisense RNA (Fig. 4c).

## Discussion

Alternative polyadenylation is a widespread mechanism of gene regulation in mammals and is often associated with specific tissue/cell types and/or developmental stages [14-17]. Previous Northern blot analyses of human fetal and adult tissues identified *MID1 *transcripts of ~7 kb, ~4.5 kb and ~3.5 kb [6,9]. Here we show that tissue-specific alternative polyadenylation in the *MID1 *gene underlies the observed size differences. Interestingly, usage of the identified polyadenylation sites appears to be determined by the choice of alternative promoters, which themselves contribute to differential *MID1 *expression [4]. In a bioinformatic approach we further found numerous putative RNA-protein interaction motifs in the *MID1 *3'UTRs, several of which turned out to be conserved between human and other species.

We found that the human *MID1 *3'UTR contains four polyadenylation sites, PAS1–PAS4. Polyadenylation at PAS1 results in a 3.5 kb transcript and usage of PAS2 leads to a 4.5 kb transcript. Due to a size difference of only 250 bp, mRNAs polyadenylated at PAS3 and PAS4 appear as a single ~7 kb band on Northern blots.

In order to differentiate between the transcripts using either PAS3 or PAS4 we hybridized a riboprobe exclusively detecting the fourth part of the 3'UTR against commercially available Northern blots. In contrast to ubiquitous expression of the 7 kb transcript detected with a probe corresponding to the *MID1 *open reading frame (Quaderi et al. 1997), we saw expression of the PAS4 7 kb transcript to be restricted to skeletal muscle and fetal liver. Hence, these experiments prove tissue restriction of the PAS4 transcripts and ubiquitous expression of the PAS3 transcripts and indicate that PAS3 is the constitutive polyadenylation signal. Remarkably, besides the ~7 kb transcript, shorter variants of ~2 kb, ~1.35 kb and ~900 bp could be observed when using the PAS4 specific riboprobe. The use of a single stranded riboprobe for Northern blot analyses excluded the possibility that these transcripts are overlapping antisense transcripts. 5'RACE showed that these transcripts are unspliced and have transcription starts which are located in the 3'UTR. Several points indicate that these are full-length transcripts. First, the overall sizes of transcripts 1 and 2 approximately match the sizes of the 2 kb and 0.9 kb Northern bands detected in the lane loaded with RNA from fetal liver (Fig. 2a and 2b). An additional smaller 0.4 kb transcript amplified by 5'RACE did not show up on the Northern blot which might be due to its low expression (Fig. 2b). Secondly, all three transcripts contain a distinct sequence motif which is found exclusively in transcription start sites derived from 3'UTRs [15], namely a triple G at the -3 to -1 position. In addition to the triple G, Carninci et al. [15] mentioned a highly conserved region located 40 to 90 bases downstream of 3'UTR transcription start sites. Concerning the *MID1 *transcripts, conservation of the +40 to +90 region is not higher than that of the remaining 3'UTR. As PAS4 is poorly conserved in other species this polyadenylation site might be human specific and therefore a high conservation of the +40 to +90 region might not be expected. Although the functions of transcripts with transcription starts in 3'UTRs are unclear it has been suggested that they might regulate downstream genes which are encoded on the opposite strand using a sense-antisense mechanism [15]. The next neighbouring gene, the CLCN4 gene, is located at a distance of ~350 kb downstream of *MID1*. As this gene is encoded on the opposite strand compared to *MID1 *such a sense-antisense regulation seems possible. On the other hand the three identified transcripts might encode short proteins. However, inspection of the sequence of transcript 1 which also contains the sequences of transcripts 2 and 3 revealed the longest protein sequence to be 83 amino acids with no conserved domains.

Tian et al. estimated that ~54% of all human genes and ~32% of all mouse genes use alternative polyadenylation sites [18]. Many human polyadenylation signals used are conserved in their rodent orthologs. Interestingly, concerning the *MID1 *polyadenylation signals, only the signal directing cleavage of PAS3 is conserved in the rat, again indicating that PAS3 is the constitutive polyadenylation site whereas PAS1, PAS2 and PAS4 can be used alternatively. This is further supported by the fact that the 7 kb transcript, which derives from transcripts using PAS3, is more strongly expressed than the 4.5 kb and 3.5 kb transcripts. Moreover, PAS3 is represented by multiple ESTs in the database that are derived from a variety of fetal and adult tissues (see additional file 1, Fig. 1a). No ESTs were found representing transcripts using PAS2 and PAS4 and only a few ESTs are present to indicate usage of PAS1.

ESTs for PAS1 are mainly derived from stomach, suggesting tissue-specific usage of PAS1.

However, a definitive statement about relative expression levels of the alternatively polyadenylated *MID1 *transcripts cannot be made at this point which is due to the following reasons: First comparison of differently sized transcripts that are detected through northern blot analyses is limited because the signal intensity is influenced by the sizes of the respective transcripts. Second the sequences of the alternatively polyadenylated *MID1 *transcripts are partially overlapping, and thus cannot be amplified individually by RT-PCR experiments.

Interestingly, we show that promoter usage is linked to poly(A) site selection in the *MID1 *gene (Fig. 3a–f). This phenomenon cannot be explained solely by expression of tissue-specific polyadenylation factors although the relative levels of expression of polyadenylation factors and transcription factors might influence the poly(A) site selection in a given cell-type [17]. Splicing factors that have a role in 3'end formation, as suggested recently [19-23] could contribute here. Also, chromatin-remodelling enzymes that can have both positive and negative roles in promoter regulation, elongation and termination could be involved [24]. In line with that hypothesis it has been suggested that predefined chromatin transcription units exist in yeast before transcription commences [24]. Furthermore, specific transcription factors that bind to both the promoter and poly(A) signal could play a role, which is supported by the observation that an increasing number of factors are essential for transcription and transcript termination [24,25]. It seems possible that all these mechanisms act together and build up a complex regulatory network that controls poly(A) site selection in order to ensure a tight control of gene expression. In the future the well characterized *MID1 *transcripts will be a suitable model for further investigation of this plausible hypothesis.

5'UTRs and 3'UTRs are implicated in the regulation of many aspects of mRNA function. 5'UTRs may contain upstream open reading frames which inhibit translation by restricting the access of ribosomes to the correct start codon [14]. Several upstream AUG codons are present in the different *MID1 *5'UTR exons and hence, it was suggested that differentially transcribed *MID1 *isoforms are translated at different levels [4]. Moreover, both 5'UTRs and 3'UTRs can contain specific sites to which regulatory RNAs or proteins bind. The composition of these sites ranges from short primary sequence elements to specific secondary structures [14,26]. Sequence analyses of the *MID1 *3'UTR revealed the existence of several cytoplasmic polyadenylation elements (CPEs). Cytoplasmic polyadenylation is a key mechanism affecting genes that are involved in synaptic plasticity and controlling mRNA translation during early development [10]. It is regulated by two *cis-*acting sequences, the CPE and the upstream element AAUAAA. Although it has been suggested that CPEs are usually located within 20–30 nucleotides upstream of the AAUAAA element, examples of mRNAs with much longer CPE-to-AAUAAA distances have been described, e.g. the CPE of C11, which resides 286 nucleotides upstream of the hexamer [27]. Of note, four of the six CPEs found in the *MID1 *3'UTR are conserved in other species (see additional file 4). Besides CPEs, the *MID1 *3'UTR contains multiple AU-rich elements (AREs) of the sequence ATTTA, several of which are conserved in other species (Fig. 4a). Like functionally relevant AREs of other genes [26], four of the conserved pentamers of the *MID1 *3'UTR are embedded in much longer AU-rich sequences (Fig. 4a). AREs are well described sequence elements to which a range of different proteins can bind, e.g. AUF1, HuR and KSRP [26,28]. These proteins can influence stability and/or translation of the respective mRNAs. In a UV-crosslink assay we could identify several proteins that bind to the ARE1 motif of the human *MID1 *3'UTR. As the sizes of the identified proteins fit quite well with the sizes of several known ARE-binding proteins, such as HuR and AUF1, they were good candidates for regulating *MID1 *expression. In an RNA-protein pulldown assay we could indeed confirm binding of these proteins to the ARE1 motif of the *MID1 *3'UTR.

## Conclusion

We found that mature mRNAs of the *MID1 *gene end at four different polyadenylation sites. The different 3'UTRs of the *MID1 *gene contain several evolutionary conserved sequence motifs, which suggests a contribution of the 3'UTRs to the mRNA stability and translation of the gene. In addition, we found that expression of the *MID1 *gene is differentially regulated by the concerted action of alternative promoters and alternative polyadenylation signals both during embryonic development and in the adult.

## Methods

### RT-PCR, 3' and 5'-RACE, Northern Blot Analysis

Total RNA from human testis was purchased from BioCat (BioCat GmbH, Heidelberg, Germany). Total RNA from fetal brain was purchased from Clontech. Total RNA from rat brain was kindly provided by Dr. Diego Walther. cDNA synthesis was performed as described previously [3]. 3 μl from a total of 25 μl cDNA was used for PCR with primers annealing to different parts of the 3'- and 5'UTRs of the *MID1 *gene (for primer sequences see additional file 6). First and nested PCRs were performed following the instructions of the Expand Long Template PCR System (Roche, Germany). PCR products were excised from the gel, purified using a Gel Extraction Kit (Qiagen, Germany), cloned into the pGEM-T Easy vector (Promega) and sequenced. 3' and 5'-Race experiments were performed as described previously [3]. Amplification of cDNA was carried out using primers that annealed to different parts of the human and rat *MID1 *3'UTRs. Primer sequences for RT-PCR, 3' and 5'-Race experiments are given in additional files 5 and 6.

Multiple-tissue Northern blots (Clontech) were hybridized with ^32^P-labeled DNA probes or riboprobes. Riboprobe nbPAS4 was synthesized by *in vitro *transcribing a PCR template corresponding to a sequence 5' of PAS4. Primer sequences are given in additional file 7. Hybridizations were carried out as described previously [3].

### In vitro transcription

^32^P-labelled cRNAs or biotin-labelled cRNAs corresponding to the sense and antisense 70–307 3'UTR human MID1 were produced using purified PCR-amplified cDNA which included the T7 Polymerase promoter sequence and T7 polymerase (Promega) according to the manufacturer's procedure. Primer sequences are given in additional file 7. In vitro transcribed probes were DNAse treated and Ethanol precipitated.

### UV crosslinking assay

HeLa cells were lysed with ultrasound and centrifuged at 12,000 × g 15 min. 4°C. Reaction mixtures containing 20 μg of protein lysate in reaction buffer (5.2 mM HEPES [pH 7.9], 50 mM KCl, 10 mM DTT, 5 mg/ml heparin, 1% glycerol, 40 μg/ml yeast tRNA) and 250.000 cpm of radiolabeled probe were incubated for 10 min. at room temperature, UV crosslinked for 10 min. in a UV Stratalinker 1800 (Stratagene) and digested with 1 U each of RNAse A and RNAse T1 for 15 min. at 37°C. Complexes were resolved by electrophoresis through SDS-10% acrylamide gels, after denaturation at 95°C for 5 min. Gels were dried and exposed to X-ray film.

### RNA-protein pulldown

HeLa cells were lysed with ultrasound and centrifuged at 12,000 × g 15 min. 4°C. Reaction mixtures containing 200 μg of protein lysate in TKM buffer (20 mM Tris [pH 7.5], 150 mM KCl, 5 mM MgCl_2_) supplemented with 1% NP40, 1 mM DTT, complete protease inhibitor cocktail (Roche), 100 U of RNasin (Promega) and 3 μg of biotin-labelled probe were incubated for 1 hour at 4°C, followed by the addition of streptavidin magnetic beads and incubation for 2 hours at 4°C. After washing and denaturation at 95°C for 5 min. proteins were resolved by electrophoresis through SDS-10% acrylamide gels. Gels were blotted on PVDF membranes and Westernblot analyses performed with antibodies directed against HuR (Santa Cruz) and AUF1 (Upstate).

### Bioinformatic analyses

We used the UCSC Genome Browser March 2006 assembly to analyse the complex structure of the 3'UTR MID1 including repeat occurrence and evolutionary sequence conservation [29]. The longest 3'UTR, which spans all the shorter transcript variants, was scanned for potential polyadenylation signals [15] and known binding motifs for RNA-binding proteins (RBP) using BioPerl [30]. For detecting polyadenylation signals we used the upstream core element AATAAA and the downstream GU or U-rich element. For detecting of CPEs and AREs we used the minimal elements TTTTAT and ATTTA which have been shown to suffice for binding of interacting proteins [26,10].

## Authors' contributions

JW supervised the work and performed the 3' and 5'-RACE, Northern Blot experiments, the UV-Assay and the RNA-protein pulldown. MK performed the RT-PCR experiments. SR and SK performed the bioinformatic analyses. RS and SS supervised the work. All authors read and approved the final manuscript.

## Supplementary Material

Additional file 1 Human ESTs indicate usage of PAS1 or PAS3. The table lists all ESTs for PAS1 and PAS3. Click here for file

Additional file 2 Poly(A) signals that are in close proximity to the alternative poly(A) sites of the human and rat *MID1 *3'UTRs. This figure shows the composition of poly(A) signals for human and rat alternative poly(A) sites. Click here for file

Additional file 3 Conservation of the hexamers AAUAAA located upstream of the alternative *MID1 *polyadenylation sites in different species. Shown is an alignment of the hexamers located upstream of the alternative *MID1 *polyadenylation sites for different mammalian and other vertebrate species. Click here for file

Additional file 4 Conservation of cytoplasmic polyadenylation elements in different species. Shown is an alignment of the cytoplasmic polyadenylation elements located in the *MID1 *3'UTR for different mammalian and other vertebrate species. Click here for file

Additional file 5 List of primers used for 3' and 5'RACE. This table provides the sequences of primers used for 3' and 5'RACE experiments. Click here for file

Additional file 6 List of primers used for RT-PCR. This table provides the sequences of primers used for RT-PCR experiments. Click here for file

Additional file 7 List of primers used for generating probes for Northern blot analysis and the UV-crosslink/RNA-protein pulldown assay. This table provides the sequences of primers used for generating probes for Northern blot analysis and UV-crosslink/RNA-protein pulldown assays Click here for file
