# Supplementary material for: Alternative polyadenylation signals and promoters act in concert to control tissue-specific expression of the Opitz Syndrome gene MID1
Source: BMC Mol Biol. 2007 Nov 15;8:105. doi: 10.1186/1471-2199-8-105 (PMC2248598; doi:10.1186/1471-2199-8-105)
Supplement: Additional file 1 — Human ESTs indicate usage of PAS1 or PAS3. The table lists all ESTs for PAS1 and PAS3. [file 1471-2199-8-105-S1.doc]

Table S1

| 3'End 3 |  |
| --- | --- |
| EST | Tissue |
| AA430123 | 8-9 week total fetus |
| AA460270 | 8-9 week total fetus |
| AA460948 | 8-9 week total fetus |
| AI383629 | 8-9 week total fetus |
| AI370698 | 8-9 week total fetus |
| AA937175 | pooled |
| AI803039 | pooled |
| AA194116 | pooled |
| AI803062 | pooled |
| AI469901 | pooled |
| AI435975 | pooled |
| AA937066 | pooled germ cell tumors |
| AA865167 | pooled germ cell tumors |
| AA843502 | parathyroid tumor |
| AA694549 | parathyroid tumor |
| AI028061 | parathyroid tumor |
| AI952806 | endometrial adenocarcinoma |
| AI262011 | adenocarcinoma |
| AI281160 | adenocarcinoma |
| AI375623 | glioblastoma |
| BQ000928 | metastatic chondrosarcoma |
| BM996100 | metastatic chondrosarcoma |
| BQ020775 | metastatic chondrosarcoma |
| CK725092 | chondrosarcoma |
| AI459977 | brain, meningioma |
| AA058518 | pregnant uterus |
| AA130805 | neuroepithelium |
| AA205361 | neuroepithelium |
| AA535152 | colon |
| AI039876 | senescent fibroblasts |
| BE967532 | brain |
| R60972 | whole brain |
| R61220 | whole brain |
| BG988219 | adult head_neck |
| BE646091 | prostate |
| BF222270 | prostate |
| BG435415 | placenta |
| BQ013939 | placenta |
| DB378950 | placenta |
| BU687647 | lung |
| BU680786 | lung |
| BU689990 | lung epithelial cells |
| CA311865 | lung epithelial cells |
| BU608621 | lung epithelial cells |
| CA311438 | lung epithelial cells |
| CB854467 | primary lung epithelial cells |
| AW242342 | kidney |
| AA026423 | fetal heart |
| W95991 | fetal heart |
| BM666313 | fetal eyes |
| N71162 | fetal lung |
| AA676704 | fetal liver + spleen |
| CX865882 | embryonic stem |

| 3'End 1 |  |
| --- | --- |
| EST | Tissue |
| AA629165 | pooled |
| AA195750 | pooled |
| AA456728 | pooled |
| AA242882 | pooled |
| BF109786 | pooled |
| AW613312 | adenocarcinoma |
| AI797530 | colon |
| AJ708093 | heart |
| AW388689 | stomach |
| AW388452 | stomach |
| AW388708 | stomach |
| AW388454 | stomach |
| AW388703 | stomach |
| AW388528 | stomach |
| AW388619 | stomach |
| AW388718 | stomach |
| AW388423 | stomach |
| AW388462 | stomach |
| AW388692 | stomach |
| AW388349 | stomach |
| AW388301 | stomach |
| BF374807 | stomach |
| BM804707 | ovary |
