# Supplementary material for: Alternative polyadenylation signals and promoters act in concert to control tissue-specific expression of the Opitz Syndrome gene MID1
Source: BMC Mol Biol. 2007 Nov 15;8:105. doi: 10.1186/1471-2199-8-105 (PMC2248598; doi:10.1186/1471-2199-8-105)
Supplement: Additional file 2 — Poly(A) signals that are in close proximity to the alternative poly(A) sites of the human and rat MID1 3'UTRs. This figure shows the composition of poly(A) signals for human and rat alternative poly(A) sites. [file 1471-2199-8-105-S2.ppt]

## Slide 1
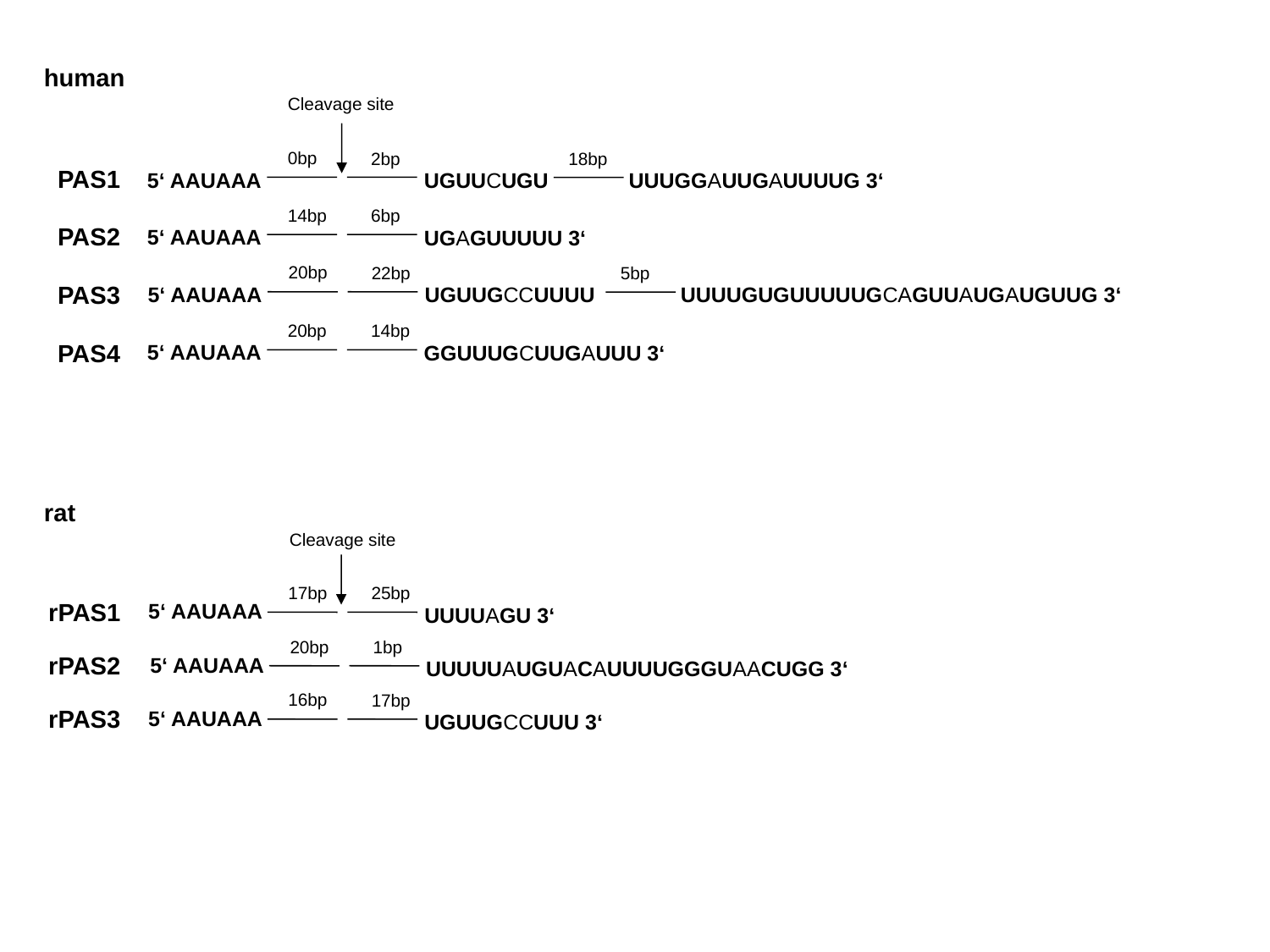

human
Cleavage site
0bp
2bp
18bp
PAS1
5‘ AAUAAA
UGUUCUGU
UUUGGAUUGAUUUUG 3‘
14bp
6bp
UGAGUUUUU 3‘
PAS2
5‘ AAUAAA
20bp
22bp
5bp
UGUUGCCUUUU
UUUUGUGUUUUUGCAGUUAUGAUGUUG 3‘
PAS3
5‘ AAUAAA
20bp
14bp
GGUUUGCUUGAUUU 3‘
PAS4
5‘ AAUAAA
rat
Cleavage site
17bp
25bp
UUUUAGU 3‘
rPAS1
5‘ AAUAAA
20bp
1bp
rPAS2
5‘ AAUAAA
UUUUUAUGUACAUUUUGGGUAACUGG 3‘
16bp
17bp
rPAS3
5‘ AAUAAA
UGUUGCCUUU 3‘
