# Supplementary material for: Alternative polyadenylation signals and promoters act in concert to control tissue-specific expression of the Opitz Syndrome gene MID1
Source: BMC Mol Biol. 2007 Nov 15;8:105. doi: 10.1186/1471-2199-8-105 (PMC2248598; doi:10.1186/1471-2199-8-105)
Supplement: Additional file 3 — Conservation of the hexamers AAUAAA located upstream of the alternative MID1 polyadenylation sites in different species. Shown is an alignment of the hexamers located upstream of the alternative MID1 polyadenylation sites for different mammalian and other vertebrate species. [file 1471-2199-8-105-S3.ppt]

## Slide 1
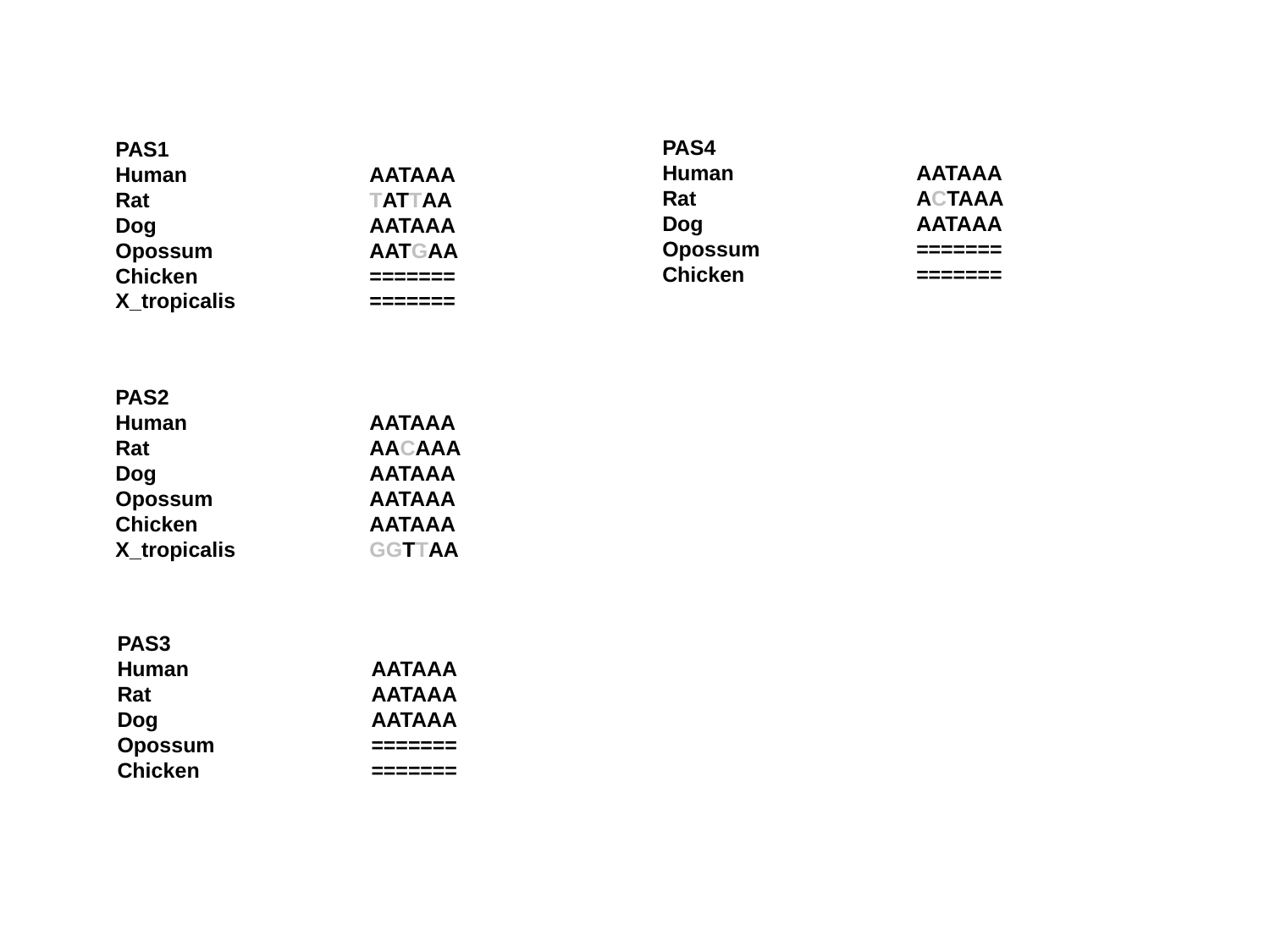

PAS4
Human		AATAAA
Rat		ACTAAA
Dog		AATAAA
Opossum		=======
Chicken		=======
PAS1
Human		AATAAA
Rat		TATTAA
Dog		AATAAA
Opossum		AATGAA
Chicken		=======
X_tropicalis		=======
PAS2
Human		AATAAA
Rat		AACAAA
Dog		AATAAA
Opossum		AATAAA
Chicken		AATAAA
X_tropicalis		GGTTAA
PAS3
Human		AATAAA
Rat		AATAAA
Dog		AATAAA
Opossum		=======
Chicken		=======
