# Supplementary material for: Alternative polyadenylation signals and promoters act in concert to control tissue-specific expression of the Opitz Syndrome gene MID1
Source: BMC Mol Biol. 2007 Nov 15;8:105. doi: 10.1186/1471-2199-8-105 (PMC2248598; doi:10.1186/1471-2199-8-105)
Supplement: Additional file 4 — Conservation of cytoplasmic polyadenylation elements in different species. Shown is an alignment of the cytoplasmic polyadenylation elements located in the MID1 3'UTR for different mammalian and other vertebrate species. [file 1471-2199-8-105-S4.ppt]

## Slide 1
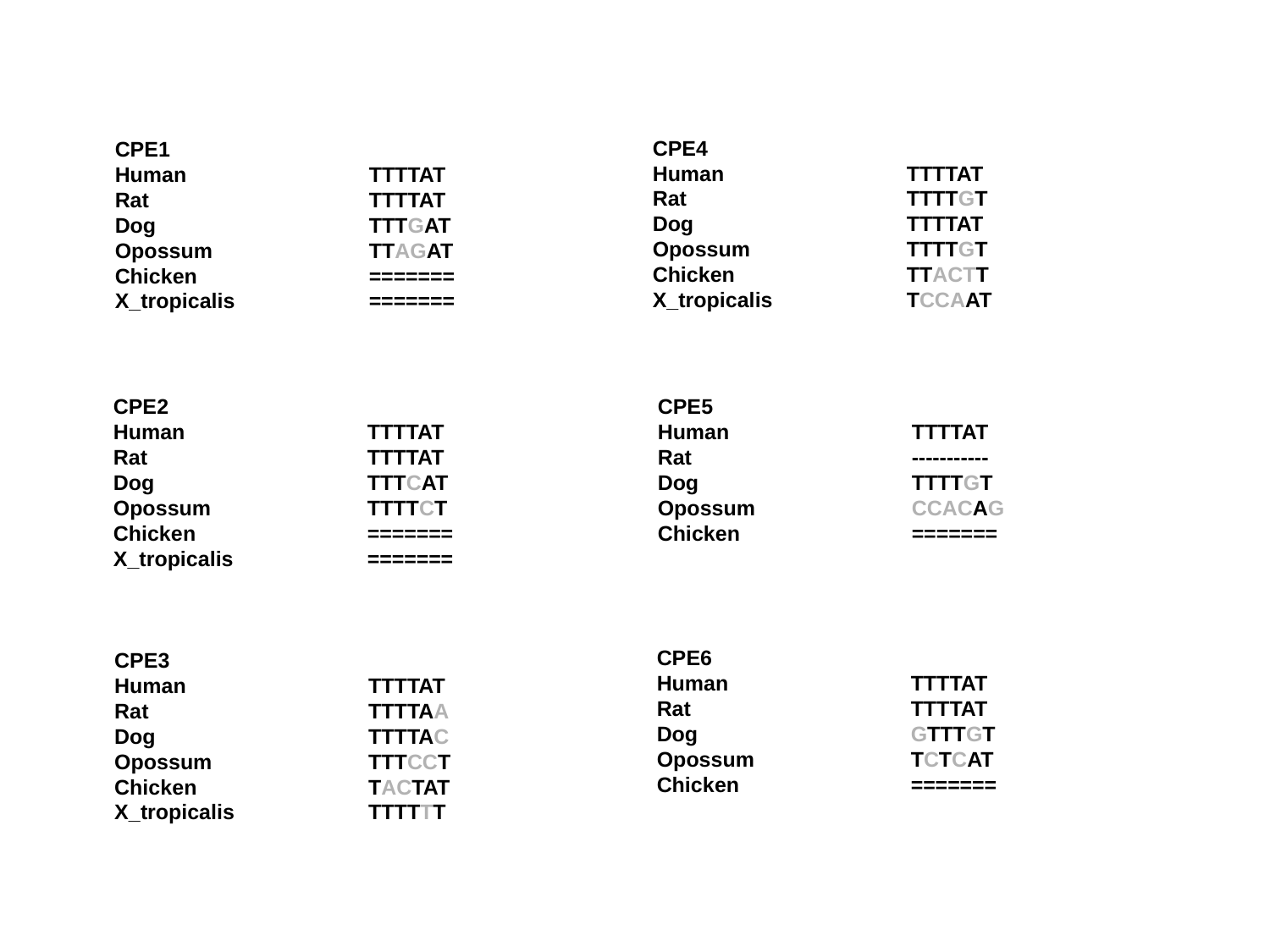

CPE4
Human		TTTTAT
Rat		TTTTGT
Dog		TTTTAT
Opossum		TTTTGT
Chicken		TTACTT
X_tropicalis		TCCAAT
CPE1
Human		TTTTAT
Rat		TTTTAT
Dog		TTTGAT
Opossum		TTAGAT
Chicken		=======
X_tropicalis		=======
CPE2
Human		TTTTAT
Rat		TTTTAT
Dog		TTTCAT
Opossum		TTTTCT
Chicken		=======
X_tropicalis		=======
CPE5
Human		TTTTAT
Rat		-----------
Dog		TTTTGT
Opossum		CCACAG
Chicken		=======
CPE6
Human		TTTTAT
Rat		TTTTAT
Dog		GTTTGT
Opossum		TCTCAT
Chicken		=======
CPE3
Human		TTTTAT
Rat		TTTTAA
Dog		TTTTAC
Opossum		TTTCCT
Chicken		TACTAT
X_tropicalis		TTTTTT
