# Supplementary material for: Alternative polyadenylation signals and promoters act in concert to control tissue-specific expression of the Opitz Syndrome gene MID1
Source: BMC Mol Biol. 2007 Nov 15;8:105. doi: 10.1186/1471-2199-8-105 (PMC2248598; doi:10.1186/1471-2199-8-105)
Supplement: Additional file 5 — List of primers used for 3' and 5'RACE. This table provides the sequences of primers used for 3' and 5'RACE experiments. [file 1471-2199-8-105-S5.doc]

Table S2. Primers (5’ 3’) 3’ and 5’Race experiments

| experiment | Forward 1.PCR | Reverse 1.PCR | Forward nested PCR | Reverse nested PCR | species |
| --- | --- | --- | --- | --- | --- |
| 3’Race h1 | cctgcttctctcagggttaagggttctg | UPM (Clontech) | gggttctggaagaacattaag | NUP (Clontech) | Human |
| 3’Race h2 | cagctgggattctttgcatgccaatctg | UPM (Clontech) | Ttgatgaagggcatgacctc | NUP (Clontech) | Human |
| 3’Race h3 | cacggtctccaggtaccaataaatgctac | UPM (Clontech) | Tgtggaagtctcactcttgg | NUP (Clontech) | Human |
| 3’Race h4 | ctctgcctgtctctgttgccttttcatc | UPM (Clontech) | ctcatccactatttgcctttcc | NUP (Clontech) | Human |
| 3’Race r1 | ccaacttgtccagaaaccagctcagatag | UPM (Clontech) | Agctcagatagggctccaca | NUP (Clontech) | Rat |
| 3’Race r2 | ggacattgacatgggttcaccttgataa | UPM (Clontech) | Ggattgaggcattggttttg | NUP (Clontech) | Rat |
| 3’Race r3 | agagagacatgtgactgcctgttcagtg | UPM (Clontech) | aaacatcaatggcatgcaga | NUP (Clontech) | Rat |
| 5’Race PAS4 | gggaaaggcaaatagtggatgagagacag | UPM (Clontech) | gagagacagtaaaagaaaatg | NUP (Clontech) | Human |
