# Supplementary material for: Alternative polyadenylation signals and promoters act in concert to control tissue-specific expression of the Opitz Syndrome gene MID1
Source: BMC Mol Biol. 2007 Nov 15;8:105. doi: 10.1186/1471-2199-8-105 (PMC2248598; doi:10.1186/1471-2199-8-105)
Supplement: Additional file 6 — List of primers used for RT-PCR. This table provides the sequences of primers used for RT-PCR experiments. [file 1471-2199-8-105-S6.doc]

Table S3. Primers (5’ 3’) RT-PCR experiments

| Exon/primer | For/Rev | 1.PCR | nested |
| --- | --- | --- | --- |
| Exon v1a | Forward | Actgagaggcgtgtaagcac | taagcacacaccctggagag |
| Exon 1c | Forward | Gggtcatcgggattctaaac | gggattctaaacatgaggcag |
| Exon 1e | Forward | Ctgtctatcatttcgtgggtc | gaactagtgtgcagtccattg |
| Primer set 1 | Reverse | Agccctatctgagccgattt | Atttcgggacacttctggtg |
| Primer set 2 | Reverse | Gaaggcaaacacacattgctt | Cattgaggcgtccatctttt |
| Primer set 3 | Reverse | Tggtgctgcattgattttgt | ccatctcttttgcccctgta |
