# Supplementary material for: Alternative polyadenylation signals and promoters act in concert to control tissue-specific expression of the Opitz Syndrome gene MID1
Source: BMC Mol Biol. 2007 Nov 15;8:105. doi: 10.1186/1471-2199-8-105 (PMC2248598; doi:10.1186/1471-2199-8-105)
Supplement: Additional file 7 — List of primers used for generating probes for Northern blot analysis and the UV-crosslink/RNA-protein pulldown assay. This table provides the sequences of primers used for generating probes for Northern blot analysis and UV-crosslink/RNA-protein pulldown assays [file 1471-2199-8-105-S7.doc]

Table S4. Primers (5’ 3’) probes

| Probe | Forward | Reverse | Nested forward | Nested reverse |
| --- | --- | --- | --- | --- |
| Ish1 | Ctgtgatgagtgcctgaaag | ggcatggtcattctccttca | Tacaggccatcgtctgattg | cagtgagtgttctgcttggga |
| Ish8 | Agcagccccaaggaattaca | ccaagtgattcctaacagctg | No nested PCR | No nested PCR |
| Ish9 | Gcagttactatttccaggttg | taatatgcaaggcccagtgc | No nested PCR | No nested PCR |
| Exon 1c | Gcttccctgcgaccgacttttc | Ctgcctcatgtttagaatccc | No nested PCR | No nested PCR |
| Exon 1e | Acagacacacaggaccgctc | Caatggactgcacactagttc | No nested PCR | No nested PCR |
| nb PAS4 | caccaagaccctctgtctct | cctcaccccatctataagac | caccaagaccctctgtctct | Ccaagcttctaatacgactcattaggga-  gaaccttgttctgggtttccac |
| UV-Assay sense | ccaagcttctaatacgactcactatagggagagcacaggcttcatgagtgta | cttatgggcctcttaatgtgc |  |  |
| UV-Assay antisense | gcacaggcttcatgagtgta | ccaagcttctaatacgactcactatagggagacttatgggcctcttaatgtgc |  |  |
